# Factors relevant to work participation from the perspective of adults with developmental dyslexia: a systematic review of qualitative studies

**DOI:** 10.1186/s12889-022-13436-x

**Published:** 2022-05-31

**Authors:** Joost de Beer, Yvonne Heerkens, Josephine Engels, Jac van der Klink

**Affiliations:** 1 Tranzo, Scientific Center for Care and Wellbeing, Tilburg School of Social and Behavioral Sciences, Tilburg University, Tilburg, the Netherlands; 2 Department Occupation & Health, HAN University of Applied Sciences, Nijmegen, The Netherlands; 3 Optentia, North-West University of South Africa, Vanderbijlpark, South Africa

**Keywords:** Adult, ICF, Developmental dyslexia, Work participation, Neurodiversity

## Abstract

**Background:**

This review is focused on workers with developmental dyslexia (DD). In this review DD is considered an expression of neurodiversity, a consequence of a natural variant of the brain. Evidence was synthesized to explore which factors workers with DD consider relevant for their participation in work and whether these factors reflect shifts in the concepts of health and sustainable employability. The factors were classified according to the International Classification of Functioning, Disability and Health (ICF), adapted for occupational health.

**Methods:**

A systematic review of qualitative studies was performed. Two search strings were used to determine the population and the context of work. The factors were classified using a recently proposed rearrangement of the ICF scheme that places participation in a central position and incorporates preliminary lists of work-related environmental factors and personal factors.

**Results:**

Fifty-one factors were found that appeared in 35% or more of the included studies and that were relevant to work participation according to the workers themselves. These factors were dispersed over all ICF categories. In the category Functions and Structures (11 factors), most of the factors had negative connotations. In the category Activities (9 factors), all the factors cause difficulties, except speaking (which is ambiguous). In the category Participation (4 factors), the formal relationships are important for the degree of participation. Overall, more than half of the factors are environmental (18) or personal (9) and they both hinder and facilitate work participation.

**Conclusions:**

The results of this review give an indication for the importance of the biopsychosocial model as a relevant approach for people with a disability in the world of work. This review also adds data for the usefulness of the proposals for the reconsideration of the ICF scheme. The data has not (yet) returned any visible trends revealing that the concept of neurodiversity is common in organizations.

**Supplementary Information:**

The online version contains supplementary material available at 10.1186/s12889-022-13436-x.

## Background

In 2014 a systematic review of qualitative and quantitative studies was performed by the same authors as the present review [1]. The aim of the review was to determine factors that hinder and facilitate work participation of people with developmental dyslexia (DD). The factors were classified according to the dimensions in the International Classification of Functioning, Disability and Health (ICF) [2]. The most important finding in that review was that DD affects not only activities related to writing/spelling and reading, but also many other activities as well as mental functions and participation. The extent of the impact of DD is influenced by many environmental and personal factors and increases over a person’s lifetime with consequences for finding and/or maintaining meaningful work.

The first rationale for performing this review is to update the former review of 2014. In that review the influencing factors for work participation were searched in both qualitative and quantitative studies and from the perspectives of the workers with DD themselves and of the employers. This review has a sharper focus: only on workers with DD themselves and the influencing factors were searched only in qualitative studies. This choice was motivated by the broadened focus in the field of occupational health to capture the complex relationship between the working environment and health in general. That makes it necessary to understand people’s experiences, behaviors, and interactions by interpreting their lived reality. Such an interpretation can provide information about a wide range of barriers to and facilitators of working with a chronic condition, such as DD, that are difficult to capture in quantitative data [3].

Another rationale for performing this review is changes in the ICF scheme. As in the 2014 review, this review used the ICF to classify the influencing factors. However, a reconsideration of the ICF scheme has been ongoing since 2017. While the original scheme placed the component ‘disease’ at the top, Heerkens et al. [4] have proposed rearranging this scheme by accommodating the disease in the component ‘personal factors’ and by giving the component ‘participation’ a central position.

Heerkens et al. [5] also elaborated on the contextual factors for occupational health care which resulted in a preliminary list of work-related environmental factors and personal factors. Both preliminary lists and the rearrangement of the ICF scheme were used in this review.

These changes and elaborations of the ICF are a result of some developments related to work and health. In the domain of health, there has been a shift from the biomedical toward a biopsychosocial paradigm, and from cure toward care, prevention, and a focus on functioning [4]. More attention is being paid to a disorder’s impact on functioning in daily life, to the positive or negative influence of a person’s character and personality traits, and to the influence of the environment (home, school/work, sports, neighborhood).

In this review DD is considered to be an expression of neurodiversity, a consequence of a natural variant of the brain [6]. Disorders that were formerly called learning or behavioral disorders (e.g., DD, dyscalculia, Attention Deficit (Hyperactivity) Disorder, Autism Spectrum Disorders, and high giftedness) are now seen as consequences of natural variants of the brain [7]. These variants can be diagnosed increasingly accurately due to the improved quality of measurement instruments. Many of these variants, like DD, are therapy-resistant and persist in adulthood [8]. Improvements in educational systems also have ensured that more attention is paid to the special needs of children and adolescents with one or more of these natural brain variants.

These factors have increased the prevalence of workers with one or a combination of these variants in recent decades [9] and have originated the concept of neurodiversity in the workforce. Advocates of neurodiversity promote a shift from complaints toward strengths [6] and assert that companies would benefit from recognizing and developing the strengths of workers with e.g. DD instead of pathologizing their weaknesses. While neurodiverse workers experience difficulties, they also bring talents to a company, such as ‘out-of-the-box thinking’ skills that balance ‘regular thinking’ skills, already present to a great extent, and different views on reality. This increases workplace diversity. But recognizing and developing these strengths requires occupational accommodations that enable such employees to access their strengths and alleviate difficulties in the pursuit of inclusive and sustainable employment [6, 10]. The concept of neurodiversity affects both the work environment and the workers with e.g. DD themselves [11].

A systematic review of qualitative studies was performed with the aim to explore which factors, classified according to the adapted ICF, workers with DD themselves consider to be relevant for their participation in work. The review also explores whether these factors reflect a paradigm shift toward a biopsychosocial model and explores the impact of the concept of neurodiversity.

## Methods

### Identification of studies

The relevant literature was identified by using the results of a systematic literature review from 2014 of the same authors [1] and by performing new searches in the electronic bibliographic databases Business Source Ultimate (via Ebsco), Cinahl Plus with full text (via Ebsco), Embase (via Ovid), ERIC (via Ebsco), PsycInfo (via Ebsco), PubMed and Web of Science (Core Collection). The 2014 review considered literature from 1995 (the year in which the ADA was published) to 2013. For this review, only the qualitative studies from the 2014 review were used and they were subjected to the same procedure as the newer studies. The new systematic searches in the databases included studies published from 2013 (because that year was just partly covered in the 2014 review) untill January 2021 (the date limit for the search). The searches were focused on dyslexia, employment and qualitative research.

### Terms used in search string

To determine the population the following terms were used in a controlled vocabulary and in title/abstract (tiab): dyslexia[MeSH] OR "Learning Disabilities"[Mesh] OR Dyslexi*[tiab] OR alexia*[tiab] OR alexic*[tiab] OR Word Blind*[tiab] OR Reading Disorder*[tiab] OR Reading Disabilit*[tiab] OR Learning Disabilit*[tiab] OR Academic Disabilit*[tiab] OR Learning Disorder*[tiab] OR Learning Disturbance*[tiab] OR Reading skill*[tiab] OR Spelling disorder*[tiab] OR reading difficult*[tiab] OR reading problem*[tiab] OR reading impairment*[tiab] OR Learning difficult*[tiab].

For ‘employment’ the search was optimized with the help of a medical information specialist for each database. Due to the nature of the used databases, it was necessary to adapt the different searches. This was done to keep the ratio between relevant and irrelevant results at an acceptable level. A more general search resulted in too many irrelevant studies.

For ‘qualitative research’ the search block with the same title was used unaltered, developed by a member of the Dutch Association of Information Professionals (KNVI) (L.J. Schoonmade), which can be found on their website [12]. The search strings and block were used in PubMed, but were modified for other databases in which different search terms are used. In all databases, the string and blocks were used in an AND-combination. Within the blocks the OR-combination was used. The complete strings per database can be found in Additional File 1.

### Inclusion criteria

The set used to include studies in this phase of the review consisted of four criteria:

A. Population
  1. ‘Dyslexia’ or ‘(specific) learning/reading disorder/disability’ mentioned explicitly in the title or abstract.
  2. Addressed a working population aged 18 to 65 years.
B. Method
  3. Primary research paper with a qualitative methodology, published after 2012 in English, German, or Dutch, and freely accessible through subscriptions at our institutions and interlibrary loan.
C. Outcome
  4. Focused on the relationship between dyslexia or (specific) learning/reading disorder/disability and work/employment/occupation from the perspective of the workers with DD themselves.

Studies were included if they met all four criteria. The criteria were not weighted. All studies identified in the searches were checked on duplications, using the Bramer method via Endnote. Afterwards the deduplication was checked manually by two authors (JdB and JE) independently. After this deduplication process all studies were imported into Rayyan for Systematic Reviews, and this software package was used throughout the inclusion of studies.

### Review procedure

Titles and abstracts of the studies identified through the search strategy were screened independently by two authors (JdB and JE). Studies that did meet the inclusion criteria were included without further examination of the full-text. Studies that did not meet the inclusion criteria were discarded. When the two authors disagreed, a consensus meeting was held to solve the disagreement.

After the immediate inclusion based on the information in the title or abstract, in the second step a study was selected for full-text examination if the title or abstract:

**Table:** 

•	Left it unclear whether it was a primary study;
•	Left it unclear whether the descriptor ‘people with learning disabilities’ or ‘(learning) disabled people’ included adults with dyslexia. The word ‘dyslexia’ had to be present in the Methods or Results section;
•	Left it unclear whether the population was still studying or was already employed;
•	Referred to an activity, personal factor, environmental factor or mental function without an explicit link to work

Two reviewers (JdB and JE) independently scanned the full texts of these studies to determine whether additional information clarified the uncertainties mentioned above. If any disagreement remained after a consensus meeting, a third reviewer (YH) was consulted to make the final choice to include or exclude the study. Two reviewers (JdB and JE) also conducted independently forward and backward citation searching for the included references, resulting in no new inclusions.

### Quality assessment

This systematic review of qualitative studies was designed to find conceptually rich studies, which are studies with sufficient depth for interpretation [13]. However, there is currently no established method to assess conceptual richness which, in itself, can be an indicator of quality. Thus, in this review quality assessment was performed to define conceptually rich studies.

Two reviewers (JdB and JE) independently used the nine questions about quality that can be asked about qualitative studies [14]: worth or relevance, clarity of the research question, appropriateness of the design, context or setting, sampling, data collection, data analysis, applicability of the results in similar settings, and reflexivity of the account. This set of criteria was chosen because of the reflexivity criterion: sensitivity to how the researcher and the research process shaped the collected data, including the role of prior assumptions and experiences [14]. This is in line with recent developments in qualitative research [15].

The reviewers graded each study on the criteria from this list and marked each as ‘ +  = present’, ‘- = not present’ or ‘ ±  = insufficiently described’, without passing a final judgment of the study’s quality. A minimum level of quality of at least six ‘ + ’ and one ‘ ± ’ was chosen, to achieve the same 70% criterion as in the 2014 review. If a study did not meet that threshold, it was excluded. To measure interrater reliability at the level of the criteria, Cohen’s kappa was calculated [16].

### Data extraction

For this review, study findings were extracted from the section labelled ‘Results’ or ‘Findings’. Findings were also extracted from the ‘Abstract’ sections because findings in abstracts of qualitative studies are not always reported in the same way as in the text [17].

All included studies were initially described based on characteristics, some of which can influence the experience of adults with DD or the impact of DD on work. These characteristics were: aim of the study, country setting (because of the national disability legislation), characteristics of participants (number, gender, age, age at diagnosis (in relation to therapy and developing coping strategies)), setting/discipline (i.e. occupations included in the study), data collection method, data analysis method, and main findings.

All included studies were then assessed in terms of factors associated with the work participation of adults with DD. A factor is a single element or a construct that the workers with DD themselves believe to have a positive or negative influence on their work participation. A factor can be embedded in a quote from a participant (a first-order construct according to Schutz [18]) or in an interpretation by the researcher in the study (a second-order construct). The factors had to be mentioned explicitly in the text.

### Data classification

**Fig. 1:**
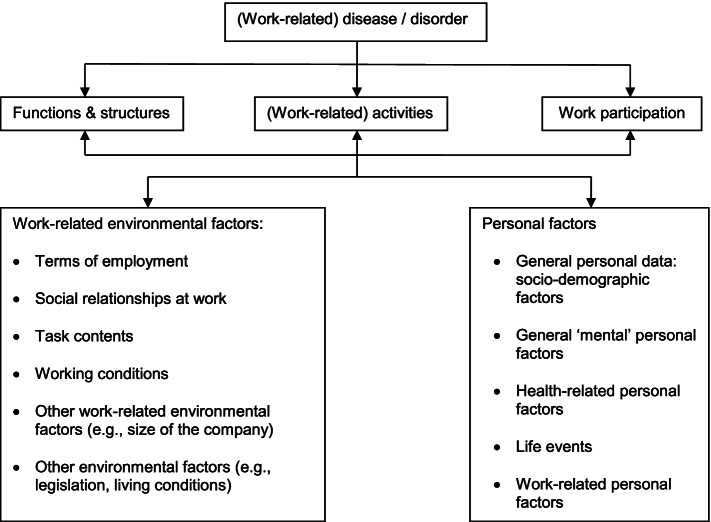
The ICF scheme, expanded with the preliminary lists of work-related environmental factors and personal factors [5]

All factors were classified according to the ICF [2]. However, the ICF does not yet classify personal factors and many factors relevant to the working environment are missing in the classification of the environmental factors. Therefore, we used the elaboration of the contextual factors for occupational health care from Heerkens et al. [5] to classify the work-related environmental factors and personal factors. The concepts used are shown in Fig. 1. The factors were linked to the best fitting ICF category or best fitting contextual factor. Although the central issue in this review is ‘work participation’, factors influencing work participation can be found in all ICF categories.

Two reviewers (JdB and YH) independently performed the data extraction and classification. Consensus meetings were part of this process. If a reviewer was uncertain about a factor or classification, a third reviewer (JE or JvdK) was consulted.

To visualize all the factors from the primary studies, the reviewers created an Excel spreadsheet with the ICF categories and preliminary lists on the y-axis and the primary studies, in chronological order, on the x-axis. More details and the spreadsheet itself are available in Additional File 2.

When evaluating the shift from a biomedical toward a biopsychosocial paradigm, the reviewers used the elaborations from the ICF and assigned a prominent position to the personal and environmental factors. The concept of neurodiversity was based on positive self-perceptions of workers with DD, a focus on strengths, positive attitudes of co-workers and line managers or employers, and support and accommodations in the workplace. Factors in the ICF related to these pillars of the neurodiversity concept can be found in the emotional functions (b152) and in the experience of self and time functions (b180); in individual attitudes of colleagues (e 325) and of people in position of authority (e430); and in products and technology for communication (e125) and for employment (e135).

## Results

In the 2014 review 13 qualitative studies were included that reached the 70% threshold for the quality assessment. For this review, the full texts of these 13 studies were independently rescreened for eligibility based on the four criteria in the Methods section. Of these 13 studies one [19] was excluded because the data were not reported from the perspective of workers with DD themselves, but from the perspective of employers.

**Fig. 2:**
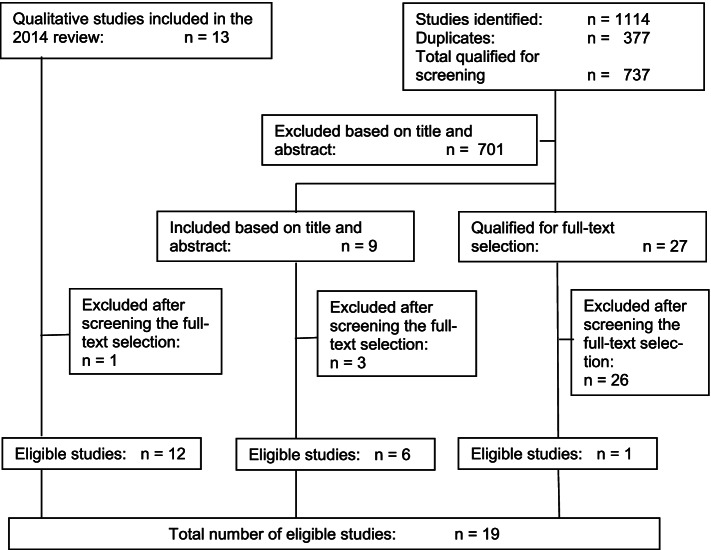
Selection of eligible studies

The additional database searches for qualitative studies on the subject from 2013 to 2021 yielded 1114 studies, 377 of which were duplicates. 737 were qualified for independent screening and were imported into Rayyan Systematic Review Software. After independent screening of title and abstract 701 studies were excluded. The remaining 36 studies required full text scrutiny: 27 had insufficient information in the title and abstract to warrant inclusion or exclusion, and 9 seemed to be eligible. These 36 studies were independently screened on the four criteria from the Methods section. Of the 27 studies with too little information in the title and abstract, 26 were excluded and 1 was included. Of the nine studies that seemed to be eligible, three were excluded and six were included. Thus the final total was 19 studies (see Fig. 2).

### Quality assessment

The nine criteria for quality reported by Mays and Pope [14] were used to assess the quality of the studies. Each study was assessed based on each criterion and assigned ‘ +  = present’, ‘- = not present’ or ‘ ±  = insufficiently described’. The threshold for inclusion in this review was at least six ‘ + ’ and one ‘ ± ’. The studies were sorted by number of plusses; those with an equal number of plusses were sorted further by publication year and alphabetical order (see Table 1). Interrater reliability at the level of the criteria independently scored by the two reviewers was measured. For that purpose a Cohen’s Kappa was calculated: 0,79, which is substantial [20].

**Table 1:** Quality assessment of included studies ranked according to quality

Study	Rele-vance	Research question	Design	Context	Sam-pling	Data collection	Data analysis	Results	Reflexi-vity	Number of + / ±
Price et al., 2003 [21]	** + **	** + **	** + **	** + **	** + **	** + **	** + **	** + **	** ± **	8/1
Yeowell et al., 2018 [22]	** + **	** + **	** + **	** + **	** + **	** + **	** + **	** ± **	** + **	8/1
McNulty, 2003 [23]	** + **	** + **	** + **	**-**	** + **	** + **	** + **	** + **	** + **	8/0
Burns and Bell, 2011 [24]	** + **	** + **	** + **	** + **	** + **	** + **	** + **	** + **	**-**	8/0
Gerber et al., 2004 [25]	** + **	** + **	** + **	** + **	** ± **	** + **	** + **	** + **	**-**	7/1
Ferri et al., 2005 [26]	** + **	** + **	** + **	** + **	**-**	** ± **	** + **	** + **	** + **	7/1
Burns et al., 2013 [27]	** + **	** + **	** + **	** + **	** + **	** + **	** ± **	** + **	**-**	7/1
Hellendoorn and Ruijssenaars, 2000 [28]	** + **	** + **	** + **	**-**	** + **	** + **	** + **	** + **	**-**	7/0
Ferri et al., 2001 [29]	** + **	** + **	** + **	** + **	** + **	** + **	**-**	** + **	**-**	7/0
Sang et al., 2016 [30]	** + **	** + **	** + **	** + **	** + **	** + **	**-**	** + **	**-**	7/0
Major and Tetley, 2019^a^ [31]	** + **	** + **	** + **	** ± **	** ± **	** + **	** ± **	** + **	** + **	6/3
Major and Tetley, 2019^b^ [32]	** + **	** + **	** + **	** + **	** ± **	** + **	** ± **	** + **	**-**	6/2
Raskind et al., 1997 [33]	** + **	** + **	** + **	**-**	**-**	** + **	** ± **	** + **	** + **	6/1
Shessel and Reiff, 1999 [34]	** + **	** + **	** + **	** + **	** ± **	** + **	**-**	** + **	**-**	6/1
Lindstrom and Benz, 2002 [35]	** + **	** + **	** + **	**-**	** ± **	** + **	** + **	** + **	**-**	6/1
Macdonald, 2009 [36]	** + **	** + **	** + **	** + **	** + **	** ± **	**-**	** + **	**-**	6/1
Newlands, 2015 [37]	** + **	** + **	** + **	** + **	** ± **	** + **	**-**	** + **	**-**	6/1
Skinner and McGill, 2015 [38]	** + **	** + **	** + **	** + **	** + **	** ± **	**-**	** + **	**-**	6/1
Locke et al., 2017 [39]	** + **	** + **	** + **	** + **	** ± **	** + **	**-**	** + **	**-**	6/1

Studies were ranked by number of plusses: those with an equal number of plusses were sorted further by publication year and alphabetical order

### Main characteristics of the studies

Table 2 displays the main characteristics of each included study: aim of the study, country, characteristics of participants, setting/discipline, data collection method, data analysis method and main findings.

**Table 2:** Main characteristics of included studies

Study	Aim of the study	Country	Participants				Setting / Discipline	Data collec-tion method	Data analysis method	Main findings
			**N**	**Gen-der**	**Age**	**Age at Diagnosis**				
Raskind et al., 1997 [33]	To learn about assistive technology from its users and discover how it can be used to compensate for LD in an employment setting	US	5	4 M 1 F	32–60	3 in adulthood, 2 as school-aged children	Currently employed in ‘white collar’ positions	Semi-structured, face-to-face and telephone interviews	Thematic analysis	Technology has been used to compensate for LD in the workplace in a wide range of ways
Shessel and Reiff, 1999 [34]	To identify and further understand the positive and negative impacts and outcomes of LD in adulthood	Canada	14	6 M 8 F	26–60	NS	Participants held a wide range of occupations	Two in-depth interviews	Inductive thematic coding in a summary to which participants were asked to respond	Negative effects and outcomes (daily living issues; the imposter phenomenon; social isolation and social perception); Positive effects and outcomes
Hellendoorn and Ruijssenaars, 2000 [28]	To identify how adults with dyslexia experience their disability, how they grew up with it and live and cope with it in their personal and working lives. To learn how dyslexia has affected their sense of self and how it has influenced their socio-emotional development	The Nether-lands	27	12 M 15 F	20–39	The age of first definite diagnosis varied considerably	Participants' occupations varied considerably	Open in-depth interviews	According to criteria outlined by Glaser and Strauss (1967)	Acceptance, openness and ways of coping; secondary education, vocational training and career experiences; experiences in the socioemotional domain; self-concept
Ferri et al., 2001 [29]	To draw out the stories of a group of adults with LD who can tell us first-hand how they experienced special education, first as students receiving services and now as teachers providing services to others	US (south central metropolitan area)	3	2 M 1 F	28–29	6–8	Three teachers in elementary, middle and high schools	In-depth semi-structured interviews	Constant comparative method (Glaser and Strauss, 1967)	Disempowering expectations: low expectations and the belief that too much help can be disempowering to students with LD; LD from deficit to teaching tool
Lindstrom and Benz, 2002 [35]	To examine the factors that influence the career development process for young women with learning disabilities entering the workforce	US	6	6 F	19–21	5–11	Participants had six different occupations	In-depth open-ended interviews	A two-step coding process (Miles and Huberman, 1994): descriptive codes per case and cross-case analysis	A high level of individual motivation and personal determination; family support and advocacy; opportunities for career exploration; vocational training; supportive workplace environments
McNulty, 2003 [23]	To understand how life stories can unfold and how they might offer potential to help parents and professionals intervene in a more sensitive, supportive and effective manner	US and Canada	12	8 M 4 F	25–45	Prior to age 14	Current occupations varied considerably	Life story interviews	Narrative analysis (Atkinson, 1998)	Contending with the LD and the sense that ‘something’s wrong with me’; finding a niche in adolescence and young adulthood; resolutions and four adult ways of life; integrating the emotional experience in adult life
Price et al., 2003 [21]	To determine the issues employees with LD have in relation to job acquisition, job advancement, self-disclosure and experiences with employer attitudes and beliefs	US (New Jersey)	25	17 M 8 F	19–32	All participants were diagnosed in K-12 settings	Participants were employed in many occupations	Face-to-face interviews with a semi-structured protocol	Constant comparative analysis, postulated by Miles and Huberman (1994) and Lincoln and Guba (1985)	Job acquisition; experiences on the job; job advancement; employer perceptions about LD; self-disclosure
Gerber et al., 2004 [25]	To explore what differences (if any) there are between US and Canadian workplaces for adults with LD	US (New Jersey) and Canada (Ontario)	49	29 M 20 F	18–45	During their school years or college	Employment experiences varied widely	Face-to-face interviews	Constant comparative analysis, postulated by Miles and Huberman (1994) and Lincoln and Guba (1985)	Getting a job (assistance from family and friends; interviewing varies widely in the workplace; self-disclosure and one’s first job; requesting accommodations prior to employment); Experiences on the job (requesting and using accommodations; employer reactions to disclosure; coworker reactions to LD); Job advancement (ramifications of LD)
Ferri et al., 2005 [26]	To examine how four teachers with LD negotiate multiple, complex, and sometimes contradictory discourses about disabilities in constructing their own understanding of LD	US	4	3 M 1 F	23–46	Self-identified as having been labeled with LD	K-12 special education teachers	Three in-depth interviews	A combination of narrative and critical discourse analysis	Individuals assign constantly shifting meanings to LD based on the influence of four sources of information: cultural scripts, official discourse, personal narratives and teaching experiences. Individuals constantly negotiate these four sources in their evolving understanding of LD
Macdonald, 2009 [36]	To investigate the effects of disabling barriers on education and employment for people with dyslexia, and to learn how disabling barriers and social class structures affect the lives of people with dyslexia	UK	13	6 M 7 F	19–54	5–43	Represent a continuum of individuals from different social backgrounds	Three life story interviews	Narrative analysis	Educational narratives and disabling barriers; Disabling barriers and issues of social class within special needs education; employment narratives, disabling barriers and social class
Burns and Bell, 2011 [24]	To discover what kind of narrative resources can be identified in interviews when teachers reveal their experiences of what it is like to teach with dyslexia. To learn how the identified narrative resources are used in the narrative construction of teacher identity	England and Finland	8	5 M 3 F	Late 30 s to late 50 s	NS	All were employed in tertiary education for less than one year to more than 30 years	Narrative interviews	The analysis applied the holistic dimensions and the categorical-content approach (Lieblich et al., 1998)	Subject positions as narrative resources; emerging professional teacher identities: the sensitive and empathetic teacher, the teacher capitalizing on personal strengths, the perseverant and proactive teacher
Burns et al., 2013 [27]	To increase understanding of how teachers with dyslexia in tertiary education have developed and employed resilience strategies to deal with the challenges they face in work contexts	Finland	6	3 M 3 F	Mid 30 s-late 50 s	In adulthood	Teachers in further or higher education	Narrative interviews	Thematic analysis (Braun and Clarke, 2006)	Task-related strategies; strategies for personalizing work contexts; social support networks; nurturing self-esteem and self-efficacy
Newlands, 2015 [37]	To develop an understanding of the challenges faced by doctors with dyslexia in the first year of practice and their support requirements	Scotland	7	1 M 6 F	23–31	7–20	Year 1 doctors at Scottish hospitals	Semi-structured telephone interviews	Inductive thematic coding, in sympathy with grounded theory	Year 1 doctors indicated that due to their dyslexia, they experience difficulty with all forms of communication, time management and anxiety. There were concerns about disclosure of their dyslexia to colleagues and supervisors. Frequently used coping strategies were safety-netting and planning; technology solutions did offer some assistance
Skinner and McGill, 2015 [38]	To explore the intersection of dyslexia, paid work and the mothering of children of school age or below	Great Britain	10	10 F	20 s-40 s	NS	Details about professions were not given to preserve anonymity	Life story and semi-structured interviews	Thematic analysis	When work, dyslexia and mothering intersect; supportive and unsupportive managers; perceived positive work impacts of dyslexia and becoming a mother; what they found helpful to maintain paid work; help in the work environment; help outside of work: partner and parent involvement
Sang et al., 2016 [30]	To explore the lived experiences of men and women who work in a sector traditionally dominated by men: the transport industry	UK	19	17 M 2 F	NS	NS	UK transport industry	Focus groups and semi-structured interviews	Template analysis (Brooks et al., 2015)	Career experiences and progression; homosociality (how men uphold their dominance in society) and humor; public–private divide; the changing organization of work; constructing difference
Locke et al., 2017 [39]	To look at the effects of dyslexia on clinical practice and the coping strategies doctors use to minimize them	UK	14	NS	NS	In practice, diagnosis happened at different stages of their work and careers	Hospital- or community- based health care	Semi-structured interviews, online survey and interviews ‘in situ’	Thematic analysis	Difficulties experienced relating to dyslexia; effective workarounds
Yeowell et al., 2018 [22]	To explore the disclosure decisions physiotherapy staff with a specific learning difficulty (SpLD) make in the workplace	UK	8	4 M 4 F	NS	NS	Physiotherapy. The mean number of years as a qualified physiotherapist was 4.5 years	In-depth semi-structured interviews	Thematic analysis	Disclosing when applying for a job; positive effects of the disabled people scheme; disclosing in the workplace
Major and Tetley, 2019^a^ [31]	To identify how dyslexia might affect registered nurses, with a particular focus on practice	Great Britain and the UK	14	3 M 11 F	25–54	7–49	A broad area of nursing practice	In-depth semi-structured interviews	Template analysis	Career choices; decision to disclose; effect on practice; compensatory strategies; support from others
Major and Tetley, 2019^b^ [32]	To identify how dyslexia might affect registered nurses’ engagement in lifelong learning and how lecturers can support them	Channel Islands and UK	14	3 M 11 F	25–54	7–49	A broad area of nursing practice	In-depth semi-structured interviews	Template analysis	Recognition of dyslexia; impact of previous learning experiences; teaching and learning strategies; reasonable adjustments

Studies are listed chronologically

*NS* Not specified

The aims of these studies correspond with their qualitative character: they report the experiences, understandings, and impact of DD on the work of workers with DD in general, but sometimes also in specific contexts like nursing, medicine, physiotherapy, education, or transportation. One study [33] explored how technology can be supportive in the workplace, one [35] examined the impact of learning disabilities on young women’s career development, one [38] explored the intersection of dyslexia, paid work, and mothering, and one [25] explored the differences between US and Canadian workplaces for adults with learning disabilities after protective legislation was introduced.

Studies were performed in various countries, and some studies included participants from more than one country. Nine were (partially) performed in the UK [22, 24, 30–32, 36–39], seven in the US [21, 23, 25, 26, 29, 33, 35], three in Canada [23, 25, 34], two in Finland [24, 27] and one in the Netherlands [28]. These countries all have legislation barring discrimination of disabled people in the workplace.

The number of participants ranged from 3 to 27, with one outlier of 49 [25]. The total number of participants was 258 with an average of 13.5. One study did not specify the distribution of gender [39]; the remaining 18 studies included 123 male (50.4%) and 121 female (49.6%) participants [21–38].

The average range in ages was 24.8 – 45.4 years and three studies had narrow age ranges (28–29; 19–21; 23–31 years) [29, 35, 37]. The participants’ age at diagnosis varied considerably: in ten studies they were at least partially diagnosed during the school period [21, 23, 25, 29, 31–33, 35–37], in six studies they were at least partially diagnosed in adulthood [27, 31–33, 36, 39], in one study the diagnosis was self-identified [26], in another study the age at diagnosis varied considerably [28] and in five studies the age at diagnosis was not specified [22, 24, 30, 34, 38].

The participants in these studies worked in many occupations: eight studies reported on a wide range of occupations [21, 23, 25, 28, 33–36], four were rooted in education [24, 26, 27, 29], five involved people in medical occupations (two about doctors [37, 39], two about nurses [31, 32], one about physiotherapists [22]), one looked at people in the transportation industry [30], and one study reported no occupational details to preserve anonymity [38]. The existence of clusters, such as education or health, underlied the decision to read the studies in those clusters as a block to preserve the meaning of the factors in context.

Data collection methods varied: 13 studies relied on semi-structured in-depth (face-to-face or telephone) interviews [21, 22, 25, 26, 28, 29, 31–35, 37, 39], three used life-story interviews [23, 36, 38], two used narrative interviews [24, 27] and one used a focus group [30].

Data analysis methods also varied. Seven studies used thematic analysis [22, 27, 33, 34, 37–39], four used a constant comparative method [21, 25, 28, 29], three used narrative analysis [23, 26, 36], another three used template analysis [30–32], one used a two-step process [35] and one took a categorical content approach [24].

### Extraction of factors

Additional File 2 contains the Excel spreadsheet mentioned above and describes how the factors extracted from the studies fit into the ICF scheme, in the category order shown in Fig. 1. For this paper, the findings were scaled to the second level of the ICF categories. On the first level, the ICF divides the main domains into chapters that are itemized at a second level. These second-level items are further specified into factors, mentioned in the studies. Where the word ‘ factors’ is mentioned in the text, it should be read to include both the second-level items and these more detailed factors. 374 factors were found. The number of factors per study varied from 39 [32, 35] to 94 [33].

In the category of Functions and Structures all factors but one were scored in the chapter of ‘Mental functions’. This chapter contains a diversity of second-level functions, six of which are relevant: *Dispositions and intrapersonal functions*; *Temperament and personality functions; Memory functions; Emotional functions; Higher-level cognitive functions*; and *Experience of self and time functions.* Under Temperament and personality functions *Confidence* was scored eight times [22, 23, 26, 28, 30, 31, 33, 38], always with a negative connotation like uncertainty or insecurity. Under Emotional functions, the connotations of the factors *Fear* and *Feelings* were also negative: fear of being stigmatized or laughed at [22, 28, 30, 36], fear of failure or exposure [20, 24, 25, 28, 30], feeling different [25, 28, 29, 34], inadequate or inferior [21, 25, 28, 34] or presenting a false impression [34, 37].

In the category of Activities, factors from three chapters are scored: ‘Learning and applying knowledge’ includes the negative factors *Speed of reading* [21, 27, 33, 34, 37–39] and *Speed of writing* [21, 24, 27, 31–33, 38, 39] which are consistent over the years. The two other chapters were ‘General tasks and demands’ and ‘Communication’. In the latter, the item *Speaking* had opposite connotations: speaking is challenging for some workers [30, 32, 37, 39], while it is a strength for others [31, 38].

In the category of Participation only two chapters were covered. The first is ‘Interpersonal interactions and relationships’ in which the item *Formal relationships* was exclusively used in the context of work (with colleagues [27, 30, 31, 34], supervisors [26, 30] or clients [29, 33]). The second was ‘Major life areas’ which contained two important items: *Acquiring, keeping and terminating a job* and *Remunerative employment.* In the latter the factor *Job performance* was mentioned seven times [22, 23, 25, 30, 33, 35, 39]. But context is important to this factor: DD can affect job performance very little or very much. Changes in the organization [30], timed tasks [39], or timed job demands [34, 37] can negatively influence job performance, but accommodations on the job and assistive technology [33] can enhance it. Stable job performance is an indicator of focused career development [35].

The category of Work-related environmental factors covers the first four chapters mentioned in Fig. 1. Under ‘Terms of employment’, the factor *Promotion/Job advancement* is an issue in which context is important: some workers being demoted because of their learning disability [30], while others do not seek promotion because of problems with some skills [30, 31, 38] and a need for extra time [31, 38]. These issues might restrict employment promotion. Some workers are promoted, with or without support from their supervisor, but job advancement often leads to more responsibilities [21, 25, 39] and hence more stress [23, 25, 28, 33, 34, 37, 38].

In the chapter of ‘Social relationships at work’, in the item *People in position of authority,* the factor *Role of employer-supervisor* needs some clarification. If a worker’s DD is disclosed, the employer/supervisor is a vital partner in achieving workplace success or failure [21, 36, 38]. Some workers hint at the positive role their employer/supervisor plays in making reasonable adjustments available [35], being willing to create a flexible schedule [38], or supporting them for a promotion [30]. The employer/supervisor also plays an important role in the factor *Reactions of co-workers* in the chapter of ‘Attitudes’ which includes explicit negative reactions, like bullying [22, 37], laughing behind the worker’s back [24, 29, 33], or disbelief [36]. Workers may fear stigmatization or criticism from colleagues [36] and compare themselves constantly to their colleagues, which may lead workers with DD to feel inadequate [21, 25] or give the false impression that they are less intelligent [23]. But when a worker with DD really trusts a colleague, disclosure will follow and then collaborative work and other types of support [27]. The chapters of ‘Task content’ and ‘Working conditions’ contain clear items and factors.

In the category of Personal factors the chapter of ‘General ‘mental’ personal factors’ with 27 items is mentioned in all included studies [21–39]. The item *Learning/Coping strategies* is remarkable: it contains 68 different types of strategies of which only the factor *Asking for help* reaches the seven-study threshold [21, 25, 28, 30, 31, 34, 37]. The item *Self-disclosure* (to colleagues or supervisors) often reflects a dilemma about whether to disclose DD [21, 22, 25–31, 34, 36, 37]. Personal and environmental factors play a role in that decision.

In the chapter of ‘Health-related personal factors ‘ the item *Impact of LD/dyslexia* has a vast scope [22, 25, 26, 28, 29, 31–34, 36]: DD can have a positive impact by helping the worker become a better and stronger person (self-perception). But the impact is mostly seen as negative: DD is experienced as a definite disability that affects everyday personal and family life, schooling, work, career, and practice, social isolation, and emotional health. Interestingly, nurses [31, 32] and doctors [37, 39] who work for the NHS in the UK stated that DD has little impact on their ability to do their jobs.

This Results section has described a selection of the factors from Additional File 2 that appear in seven (35%) or more of the included studies. Table 3 presents all 51 factors without specifying in which studies they appear. That information can be found in Additional File 2.

**Table 3:** Factors that appear in seven or more studies

Category and chapter	2^nd^ level items	Factors	Number of studies
FUNCTIONS AND STRUCTURES			
**b1 Mental functions**			
	Dispositions and intrapersonal functions		7
	Temperament and personality functions		11
		Confidence	8
	Memory functions		8
	Emotional functions		16
		Fear	8
		Feelings	7
		Sense of strength	7
		Shame/embarrassment	7
	Higher-level cognitive functions		8
	Experience of self and time functions		11
ACTIVITIES (d1-d6)			
**d1 Learning and applying knowledge**			
	Acquiring skills		7
	Reading		12
		Speed of reading	7
	Writing		16
		Speed of writing	8
**d2 General tasks and demands**			
	Undertaking multiple tasks		7
	Carrying out daily routine		7
**d3 Communication**			
	Speaking		12
	Writing messages		9
PARTICIPATION (d7-d9)			
**d7 Interpersonal interactions and relationships**			
	Formal relationships		7
**d8 Major life areas**			
	Acquiring, keeping and terminating a job		10
	Remunerative employment		12
		Job performance	7
ENVIRONMENTAL FACTORS			
**Work-related environmental factors: terms of employment**			8
**e3 Social relationships at work**			
	Immediate and extended family and friends		9
		Support from family and friends	9
	Acquaintances, peers, colleagues, neighbors and community members		11
		Help from colleagues / co-worker assistance / buddy / mentor	8
		Support in the workplace	8
	People in position of authority		12
		Role of employer / supervisor	8
**e4 Attitudes**			
	Individual attitudes of acquaintances, peers, colleagues, neighbors and community members		16
		Reactions of co-workers	10
	Individual attitudes of people in position of authority		12
		Negative response of employer	9
**Task content**			15
	Workload, pressure, stress		7
**Working conditions**			
	Products and technology for communication		11
		Assistive technology	10
	Products and technology for employment		8
		Accommodations on the job	8
PERSONAL FACTORS			
**General ‘mental’ personal factors**			19
	Learning / coping strategies		18
		Asking for help	7
	Self-disclosure		12
	Stress-experience / being stressed		7
**Health-related personal factors**			12
	Impact of LD / dyslexia		10
**Work-related personal factors**			14
	Successful		7

## Discussion

This systematic review of qualitative studies found that 374 factors are relevant to work participation according to workers with developmental dyslexia themselves. Of those factors, 51 (= 13.6%) appeared in seven (35%) or more of the included studies.

In the category of Functions and Structures (11 factors) factors with a negative connotation are prevalent; only a few are assigned positive traits: perseverance/persistence, a sense of strength, or visual and out-of-the-box thinking. A positive self-perception was found in only a few cases.

In the category of Activities (9 factors) all factors cause difficulties, except speaking which is ambiguous.

In the category of Participation (4 factors), the formal relationships are important for the degree of participation. More than half of the factors are environmental (18): expressing a characteristic of the job, or personal (9): focusing on personal experience.

In the category of Environmental factors the social relationships at work and the attitudes of colleagues and managers play a decisive role in achieving successful work participation. In the context of task content, the studies reported workload, work pressure, and work stress. In the context of working conditions assistive technology for communication and accommodations on the job are helpful.

In the category of Personal factors stress experience is also an important factor. Self-disclosure remains a dilemma for most of the participants and developing learning and coping strategies is crucial for work participation. People feel the impact of DD in nearly all aspects of daily life, and it can be a barrier to success.

Of the 374 factors found, 118 (31.5%) are personal and 103 (27.5%) are environmental. This distribution indicates that the biomedical model is untenable. Context matters, as do the personality traits of workers with DD. The results of this review give an indication for the importance of the biopsychosocial model as a relevant approach for people with a disability in the world of work.

This review adds data for the usefulness of the proposals Heerkens et al. [4] made about reconsidering the ICF scheme. They proposed replacing illness or disability as the central category in the ICF scheme with the concept of functioning: an overarching term for participation, activities, and functions and structures. Functioning can be influenced by and can influence personal factors, for which an extended classification needs to be developed. And this whole is surrounded by environmental factors, that have a positive and negative reciprocal influence on functioning and personal factors. This alternative scheme was used in this review and the results have produced a good description of functioning of workers with DD, in which far more than half of the factors (221 of the 374; 59%) are personal and environmental.

As stated in the 2 section, the concept of neurodiversity can be found in several places in the ICF: in the emotional functions (b152) and in the experience of self and time functions (b180); in individual attitudes of colleagues (e 325) and of people in position of authority (e430); and in products and technology for communication (e125) and for employment (e135). Looking at the factors in these ICF categories reveals no big shifts or changes: a negative self-image and perceptions still occur, as do negative reactions from co-workers and managers. On the positive side support and job accommodations are still provided.

It is striking to note that sense of strength is not listed as a factor in the studies published after 2014. A possible explanation could be decreasing awareness of their own strengths and qualities. In line with this explanation is the disappearance of self-esteem because it is determined by achievements and accomplishments and by experiencing success. Neither factor is mentioned in the studies after 2014.

The results of this review do not give an indication for an increasing awareness of neurodiversity in the world of work. Apparently, it takes some time before theoretical concepts like neurodiversity are common in the workforce.

The quality of a working life is greatly affected by social relationships at work and the attitude of co-workers and managers. This review found that people with DD often experience these factors negatively, which is in line with the position paper by Brouwers [40] who found that employers and other stakeholders in the work environment often hold negative attitudes toward people with mental health issues. Admittedly, DD is not a mental health issue, but it has similar mechanisms. Negative attitudes decrease the likelihood that workers with DD will be supported and increase the risk of stigmatization and discrimination. The systematic review by Van Beukering et al. [41] also found that health-related stigma expressed by employers and co-workers is a barrier to sustainable employment and well-being at work for people with a disability.

For many workers with DD, disclosure (reported in 12 of the 19 studies [21, 22, 25–31, 34, 36, 37]) remains a dilemma and a complicated challenge. Whether to disclose or not depends on many factors: the worker’s character and confidence, environmental safety, the line manager’s attitude and knowledge, the desire for accommodations, and a fear of bullying, stigmatization, and discrimination. Such fear causes many workers with DD to be reluctant to voluntarily disclose their disability in advance. However, disclosure is an important condition in the disability legislation programs in different countries. The reluctance to disclose impedes the intended effect of these legislation programs on inclusion [6]. Added to that can be the strong tendency of organizations “to ‘credit’ all problems that the worker encounters to him or her as an individual, and to consider him or her responsible for the solution. […..] This infers that the organization does not feel a responsibility, let alone an urgency, to change or adapt or adjust the organizational context to be mere facilitating and inclusive.” ([42], p.23). This quote also clarifies that the medical paradigm still is predominant in the eyes of employers and entrepreneurs. That paradigm is probably the biggest barrier to diversity in the workplace.

The results of this review largely mirror the results from the review the authors performed in 2014 [1]. That is unsurprising given that the qualitative studies from that review, minus one, were also used in this study.

The included studies were conducted in five different countries: the US [21, 23, 25, 26, 29, 33, 35], (parts of the) UK [22, 24, 30–32, 36–39], Canada [23, 25, 34], the Netherlands [28], and Finland [24, 27]. No new countries were added in the studies from 2014 on. Considering the distribution of factors, it seems that there are no major differences in how these countries accommodate people with DD in the workplace.

The age at diagnosis differed widely in the included studies. Six studies before 2014 reported diagnosis between 7 and 21 years of age (school and college years) [21, 23, 25, 29, 33, 35]. There was no commonality in the other studies before 2014: some included a much wider age range at diagnosis [28, 36], while other participants were only diagnosed in adulthood [27], diagnosed themselves [26], or the age at diagnosis is not specified [24, 34]. This also applies to the studies after 2014. Comparing the studies before and after 2014 on the age at diagnosis, there seems to be no difference in the distribution of factors.

Relative to the 2014 review, the distribution of negative and positive items and factors did not change in this review. The negative items and factors are most prevalent in the included studies, and the workers only occasionally emphasized their strengths. The worker’s own negative attitude toward DD remain notable.

The occupations of study participants differed between the 2014 review and this review. Eight of the 12 studies reported on in 2014 included a wide range of occupations [21, 23, 25, 28, 33–36], and education was the context in the remaining four studies [24, 26, 27, 29]. The seven studies conducted in or after 2014 added four contexts: medicine [37, 39], nursing [31, 32], physiotherapy [22], and transportation [30]. One study reported no occupational details to preserve anonymity [38]. Together with education, these four new contexts seem to include skills that are sensitive to the influence of DD: reading out loud and speaking in public and presenting (mentioned four times after 2014, but never before [30, 32, 37, 39]), writing messages (far more factors were mentioned after 2014), and discussing [21, 24, 25, 28, 33] and sensitive to emotional experiences of others (both mentioned five times before 2014, but never after [23, 24, 27, 33, 34]). These factors seem to relate to the profession under study (i.e., writing messages in healthcare professions and discussing or being sensitive in educational professions).

This review found 68 types of coping strategies (versus 39 in the 2014 review). This increase may be related to the professional contexts included in six of the seven studies after 2014: medicine [37, 39], nursing [31, 32], physiotherapy [22], and transportation [30]. Jobs in these contexts may require coping strategies to perform appropriately. In this sense, the increase in types of coping strategies could indicate increasing self-management and autonomy to minimalize the negative impact of DD on work participation. But this increase could also hint at decreasing support from the work environment (colleagues, line managers) that forces workers with DD to depend increasingly on themselves. Under such conditions, it is imaginable that workers with DD would hesitate to disclose their DD, as discussed above.

The more recent studies mention fewer factors related to the chapter of Terms of employment: there were 11 factors in five studies conducted before 2014 [21, 25, 33, 35, 36], but only four factors in three studies after 2014 [30, 31, 38]. This decrease may indicate the growing influence of disability legislation which may normalize accommodations in the work environment for workers with DD. But it could also indicate diminishing support from the work context that forces workers with DD to find their own strategies to manage the impact of DD on their work, and consequently makes them hesitant to discuss terms of employment, including asking for accommodations.

### Strengths and limitations of this review

This review used the elaborated version of the ICF for occupational health care [5]. That made it easier to identify all the factors relevant to work participation, to categorize them, and to position them in the work-related dimensions.

Only the factors seen through the eyes of the workers with DD themselves were extracted, which excluded the perspectives of the people who surround them (e.g., colleagues, line managers and employers). This focus makes it possible to capture the factors that really matter and that would be invisible in quantitative studies [3].

Also the influence of the present researchers on the construction of meanings and of lived experiences of workers with DD needs to be addressed. The data to be analyzed consisted of quotes from the workers themselves or from the researchers in the primary studies. These quotes were sometimes extensive, but they could also be very short. The literal text was used to describe the meaning of a factor, but classifying a factor into a second-level ICF item is a subjective choice and an act of interpretation. To reduce subjectivity, the analysis was done by at least two authors (JdB and YH), and, in the case of doubt, by a third author (JvdK/JE). Nevertheless, it is possible that factors mentioned in the text were incorrectly interpreted and classified in the ICF or that two distinct factors may cover the same meaning. To make the interpretations and classifications auditable, the choice for an exhaustive detailing was made in the Excel spreadsheet in Additional File 2. However, the exhaustive details negatively affected the spreadsheet’s clarity.

For the quality assessment the criteria list based on Mays and Pope [14] was chosen because of the additional criterion ‘reflexivity’. This choice reflects our assumption that in more recent studies, researchers would be more conscious of their own influence on data collection and analysis. However, that was not the case: of the seven studies after 2014 only two made remarks about reflexivity [22, 31]. That is the same proportion as in the studies before 2014 (four to twelve [21, 23, 26, 33]).

### Implications for practice

Along with Attention Deficit (Hyperactivity) Disorder (ADHD) and Autistic Spectrum Disorders (ASD) DD belongs to the ‘neurominorities’ as Doyle [6] defines them: the diversity within an individual’s cognitive ability. DD is a chronic condition, the incidence of which has been increasing in recent decades. In education, improvements have been made in coaching children and adolescents with one or more of these conditions. Thus, in the future more people from the ‘neurominorities’ will enter the labor market, but this labor market is not well prepared for their arrival: there is too little knowledge of their strengths and weaknesses, colleagues and line managers often hold stigmatizing attitudes, the workers themselves may have inadequate self-management capacities, and there is too little knowledge on how to accommodate them in the workplace and give them the right type of support. These and other barriers must be overcome. There is a need for a tool in the workplace that can be used to discuss all the relevant aspects that can be influenced and that can support an increase in work participation and job satisfaction. An overview of the relevant factors may be a starting point for constructing such a tool. A better understanding of these factors, arrived at by analyzing the possible combinations and types of relationships, will enhance the inclusion of workers with neurodiverse features in the workplace.

### Suggestions for further research

The factors mentioned in the Results section are part of a bigger network in which they can be combined with other factors in various relationships. Merely aggregating these factors, as this review has done, does not reveal those combinations and relationships. Therefore, another type of analysis is needed to provide a nuanced understanding of an issue (work participation of workers with DD) within larger theoretical, social, and cultural contexts. Meta-ethnography would provide that type of comparative textual analysis of qualitative studies [43]. Analyzing the textual material (the first- and second-order constructs) from the qualitative studies in this review in an ethnographic way, has already begun and the results will be reported in another article.

The central position of functioning in the adapted ICF scheme makes it easier to connect to the Capability Approach (CA) [44]. This first was done by Welsh Saleeby [45] and subsequently adopted by Bickenbach and Mitra [46, 47]. The CA recognizes functionings and capabilities as central concepts. The capabilities are the real possibilities to choose from, for doing or being what a person has reason to value. The functionings are the achievements of that process and they come very close to the concept of participation [48]. The ICF and the CA can complement each other: the ICF has fewer options to express individual orientation on values in life, underlying personal aspirations, and choices, while the CA needs valid and comparable data about the health status of individuals. It would be interesting to use a combined ICF–CA framework to validate the numerous factors found in this review. With that aim, qualitative in-depth interviews were performed with workers with DD, also to operationalize the value of work for these adults. The results of these interviews will be reported in another article.

## Conclusion

The results of this review give an indication for the importance of the biopsychosocial model as a relevant approach for people with disabilities in the world of work. This review also adds data for the usefulness of the proposals made by Heerkens et al. [4] about the reconsideration of the ICF scheme. The data has not (yet) returned any visible trends revealing that the concept of neurodiversity is common in organizations. As far as the increase in coping strategies is concerned, it is difficult to unambiguously interpret these results.

According to the sustainable development goals of the United Nations [49], it is a societal responsibility to employ workers with a work-related disability. This is Doyle’s [6] occupational narrative around the ‘diamond in the rough’: the aim of occupational accommodations is to access the strengths of workers with work-related disabilities like DD and to alleviate their struggles with the goal of including a great diversity of people in the workplace, which also benefits the organization itself and society as a whole.

## Supplementary Information

**Additional file 1.** Complete search strings per database

**Additional file 2.** All extracted factors, classified according to the ICF-scheme 

## Data Availability

All data generated or analyzed during this study are included in this published article [and its supplementary information files]. To obtain the data, a request can be sent to the corresponding author: joost.debeer@han.nl.
