# Supplementary material for: Factors relevant to work participation from the perspective of adults with developmental dyslexia: a systematic review of qualitative studies
Source: BMC Public Health. 2022 May 31;22:1083. doi: 10.1186/s12889-022-13436-x (PMC9158268; doi:10.1186/s12889-022-13436-x)
Supplement: Supplementary file 1 — Additional file 1. Complete search strings per database [file 12889_2022_13436_MOESM1_ESM.docx]

**Additional File 1: Complete search strings per database**

**Business Source Ultimate (via Ebsco)**

S1 TI (Dyslexi* OR alexia* OR alexic* OR (Word N1 Blind*) OR (Reading N1 Disorder*) OR (Reading N1 Disabilit*) OR (Learning N1 Disabilit*) OR (Academic N1 Disabilit*) OR (Learning N1 Disorder*) OR (Learning N1 Disturbance*) OR (Reading N1 (skill* OR ability*)) OR (Spelling N1 disorder*) OR (reading N1 difficult*) OR (reading N1 problem*) OR (reading N1 impairment*) OR (Learning N1 difficult*)) OR AB (Dyslexi* OR alexia* OR alexic* OR (Word N1 Blind*) OR (Reading N1 Disorder*) OR (Reading N1 Disabilit*) OR (Learning N1 Disabilit*) OR (Academic N1 Disabilit*) OR (Learning N1 Disorder*) OR (Learning N1 Disturbance*) OR (Reading N1 (skill* OR ability*)) OR (Spelling N1 disorder*) OR (reading N1 difficult*) OR (reading N1 problem*) OR (reading N1 impairment*) OR (Learning N1 difficult*)) OR SU (Dyslexi* OR alexia* OR alexic* OR (Word N1 Blind*) OR (Reading N1 Disorder*) OR (Reading N1 Disabilit*) OR (Learning N1 Disabilit*) OR (Academic N1 Disabilit*) OR (Learning N1 Disorder*) OR (Learning N1 Disturbance*) OR (Reading N1 (skill* OR ability*)) OR (Spelling N1 disorder*) OR (reading N1 difficult*) OR (reading N1 problem*) OR (reading N1 impairment*) OR (Learning N1 difficult*))

S2 DE "JOB absenteeism" OR DE "SICK leave" OR DE "JOB performance" OR DE "JOB satisfaction" OR DE "EMPLOYEE complaints" OR DE "JOB stress" OR DE "WORK environment" OR DE "WORK experience (Employment)" OR DE "WORK-life balance" OR DE "OCCUPATIONS" OR DE "VOCATIONAL rehabilitation" OR DE "SUPPORTED employment" OR DE "UNDEREMPLOYMENT" OR DE "UNEMPLOYMENT" OR DE "EMPLOYEES' workload" OR DE "EMPLOYEE empowerment" OR DE "LABOR mobility" OR DE "EMPLOYABILITY" OR DE "EMPLOYMENT reentry" OR DE "SELF-employment" OR DE "PART-time self-employment" OR DE "JOB applications" OR TI ((career N1 choice*) OR ((Disability OR sick) N1 Leave*) OR (employment N1 status) OR ((sick* OR Illness) N1 Day*) OR ((job* OR work* OR labor* OR labour* OR occupation* OR vocation* OR profession*) N1 (site* OR status OR stress* OR applicat* OR particip* OR location OR satisf* OR performance* OR characteristic* OR accommodation* OR experience* OR skill* OR security* OR rehab*)) OR (Job-related N1 Stress*) OR (Qualit* N1 Work* N1 Life) OR ((temporary OR “Part-time”) N1 (Job* OR Employment OR work* OR labour OR labor)) OR (work N1 life N1 balanc*) OR (work* N1 load*) OR (Work* N1 Scheduling) OR (Work* N1 place*) OR (Work-related N1 Stress*) OR Absenteeism* OR Employabilit* OR Employment* OR Job OR jobs OR jobsite* OR labor* OR labour* OR Underemploy* OR Unemploy* OR Vocational* OR Work OR Workload OR workplace* OR worksite*) OR AB ((career N1 choice*) OR ((Disability OR sick) N1 Leave*) OR (employment N1 status) OR ((sick* OR Illness) N1 Day*) OR ((job* OR work* OR labor* OR labour* OR occupation* OR vocation* OR profession*) N1 (site* OR status OR stress* OR applicat* OR particip* OR location OR satisf* OR performance* OR characteristic* OR accommodation* OR experience* OR skill* OR security* OR rehab*)) OR (Job-related N1 Stress*) OR (Qualit* N1 Work* N1 Life) OR ((temporary OR “Part-time”) N1 (Job* OR Employment OR work* OR labour OR labor)) OR (work N1 life N1 balanc*) OR (work* N1 load*) OR (Work* N1 Scheduling) OR (Work* N1 place*) OR (Work-related N1 Stress*) OR Absenteeism* OR Employabilit* OR Employment* OR jobsite* OR Underemploy* OR Unemploy* OR Workload OR workplace* OR worksite*) OR SU ((career N1 choice*) OR ((Disability OR sick) N1 Leave*) OR (employment N1 status) OR ((sick* OR Illness) N1 Day*) OR ((job* OR work* OR labor* OR labour* OR occupation* OR vocation* OR profession*) N1 (site* OR status OR stress* OR applicat* OR particip* OR location OR satisf* OR performance* OR characteristic* OR accommodation* OR experience* OR skill* OR security* OR rehab*)) OR (Job-related N1 Stress*) OR (Qualit* N1 Work* N1 Life) OR ((temporary OR “Part-time”) N1 (Job* OR Employment OR work* OR labour OR labor)) OR (work N1 life N1 balanc*) OR (work* N1 load*) OR (Work* N1 Scheduling) OR (Work* N1 place*) OR (Work-related N1 Stress*) OR Absenteeism* OR Employabilit* OR Employment* OR jobsite* OR Underemploy* OR Unemploy* OR Workload OR workplace* OR worksite*)

S3 S1 AND S2

S4 TI ((job* OR work* OR labor* OR labour* OR occupation* OR vocation* OR profession*) N1 (literacy OR illitera*))

S5 S3 OR S4

S6 DE "QUALITATIVE research" OR DE "FOCUS groups" OR DE "INTERVIEWING" OR DE "TELEPHONE interviewing" OR TI (((thematic OR content) N1 analys*) OR interview* OR (focus N1 group*) OR ethnograph* OR ethnograf* OR etnograf* OR (field N1 stud*) OR phenomenolog* OR narration* OR narrative OR (qualitative N1 (stud* OR analys* OR research* OR method*)) OR multimethodolog* OR (mixed N1 method*) OR observation* OR (grounded N1 theory) OR ((audio OR tape) N1 recording*) OR audiotape* OR ((“semi-structured” OR semistructured OR unstructured OR informal OR “in-depth” OR indepth OR face-to-face OR structured OR guide*) N3 (interview* OR discussion* OR questionnaire*))) OR AB (((thematic OR content) N1 analys*) OR (focus N1 group*) OR ethnograph* OR ethnograf* OR etnograf* OR (field N1 stud*) OR phenomenolog* OR narration* OR narrative OR (qualitative N1 (stud* OR analys* OR research* OR method*)) OR multimethodolog* OR (mixed N1 method*) OR observation* OR (grounded N1 theory) OR ((audio OR tape) N1 recording*) OR audiotape* OR ((“semi-structured” OR semistructured OR unstructured OR informal OR “in-depth” OR indepth OR face-to-face OR structured OR guide*) N3 (interview* OR discussion* OR questionnaire*))) OR SU (((thematic OR content) N1 analys*) OR (focus N1 group*) OR ethnograph* OR ethnograf* OR etnograf* OR (field N1 stud*) OR phenomenolog* OR narration* OR narrative OR (qualitative N1 (stud* OR analys* OR research* OR method*)) OR multimethodolog* OR (mixed N1 method*) OR observation* OR (grounded N1 theory) OR ((audio OR tape) N1 recording*) OR audiotape* OR ((“semi-structured” OR semistructured OR unstructured OR informal OR “in-depth” OR indepth OR face-to-face OR structured OR guide*) N3 (interview* OR discussion* OR questionnaire*)))

S7 S5 AND S6

**Cinahl ^+^ with full text (via Ebsco)**

S1 MH "Dyslexia" OR MH "Learning Disorders" OR MH "Reading Disorders+" OR TI (Dyslexi* OR alexia* OR alexic* OR (Word N1 Blind*) OR (Reading N1 Disorder*) OR (Reading N1 Disabilit*) OR (Learning N1 Disabilit*) OR (Academic N1 Disabilit*) OR (Learning N1 Disorder*) OR (Learning N1 Disturbance*) OR (Reading N1 (skill* OR ability*)) OR (Spelling N1 disorder*) OR (reading N1 difficult*) OR (reading N1 problem*) OR (reading N1 impairment*) OR (Learning N1 difficult*)) OR AB (Dyslexi* OR alexia* OR alexic* OR (Word N1 Blind*) OR (Reading N1 Disorder*) OR (Reading N1 Disabilit*) OR (Learning N1 Disabilit*) OR (Academic N1 Disabilit*) OR (Learning N1 Disorder*) OR (Learning N1 Disturbance*) OR (Reading N1 (skill* OR ability*)) OR (Spelling N1 disorder*) OR (reading N1 difficult*) OR (reading N1 problem*) OR (reading N1 impairment*) OR (Learning N1 difficult*)) OR SU (Dyslexi* OR alexia* OR alexic* OR (Word N1 Blind*) OR (Reading N1 Disorder*) OR (Reading N1 Disabilit*) OR (Learning N1 Disabilit*) OR (Academic N1 Disabilit*) OR (Learning N1 Disorder*) OR (Learning N1 Disturbance*) OR (Reading N1 (skill* OR ability*)) OR (Spelling N1 disorder*) OR (reading N1 difficult*) OR (reading N1 problem*) OR (reading N1 impairment*) OR (Learning N1 difficult*))

S2 MH "Job Satisfaction+" OR MH "Job Performance" OR MH "Job Characteristics" OR MH "Work-Life Balance" OR MH "Job Accommodation" OR MH "Employment+" OR MH "Part Time Employment" OR MH "Employment Status" OR MH "Work Experiences" OR MH "Quality of Working Life" OR MH "Job Satisfaction" OR MH "Job Experience" OR MH "Rehabilitation, Vocational+" OR MH "Occupations and Professions" OR MH "Stress, Occupational" OR MH "Unemployment" OR MH "Sick Leave" OR MH "Absenteeism" OR MH "Job Application+" OR MH "Absenteeism" OR MH "Workload" OR TI ((career N1 choice*) OR ((Disability OR sick) N1 Leave*) OR (employment N1 status) OR ((sick* OR Illness) N1 Day*) OR ((job* OR work* OR labor* OR labour* OR occupation* OR vocation* OR profession*) N1 (site* OR status OR stress* OR applicat* OR particip* OR location OR satisf* OR performance* OR characteristic* OR accommodation* OR experience* OR skill* OR security* OR rehab*)) OR (Job-related N1 Stress*) OR (Qualit* N1 Work* N1 Life) OR ((temporary OR “Part-time”) N1 (Job* OR Employment OR work* OR labour OR labor)) OR (work N1 life N1 balanc*) OR (work* N1 load*) OR (Work* N1 Scheduling) OR (Work* N1 place*) OR (Work-related N1 Stress*) OR Absenteeism* OR Employabilit* OR Employment* OR Job OR jobs OR jobsite* OR labor* OR labour* OR Underemploy* OR Unemploy* OR Vocational* OR Work OR Workload OR workplace* OR worksite*) OR AB ((career N1 choice*) OR ((Disability OR sick) N1 Leave*) OR (employment N1 status) OR ((sick* OR Illness) N1 Day*) OR ((job* OR work* OR labor* OR labour* OR occupation* OR vocation* OR profession*) N1 (site* OR status OR stress* OR applicat* OR particip* OR location OR satisf* OR performance* OR characteristic* OR accommodation* OR experience* OR skill* OR security* OR rehab*)) OR (Job-related N1 Stress*) OR (Qualit* N1 Work* N1 Life) OR ((temporary OR “Part-time”) N1 (Job* OR Employment OR work* OR labour OR labor)) OR (work N1 life N1 balanc*) OR (work* N1 load*) OR (Work* N1 Scheduling) OR (Work* N1 place*) OR (Work-related N1 Stress*) OR Absenteeism* OR Employabilit* OR Employment* OR jobsite* OR Underemploy* OR Unemploy* OR Workload OR workplace* OR worksite*) OR SU ((career N1 choice*) OR ((Disability OR sick) N1 Leave*) OR (employment N1 status) OR ((sick* OR Illness) N1 Day*) OR ((job* OR work* OR labor* OR labour* OR occupation* OR vocation* OR profession*) N1 (site* OR status OR stress* OR applicat* OR particip* OR location OR satisf* OR performance* OR characteristic* OR accommodation* OR experience* OR skill* OR security* OR rehab*)) OR (Job-related N1 Stress*) OR (Qualit* N1 Work* N1 Life) OR ((temporary OR “Part-time”) N1 (Job* OR Employment OR work* OR labour OR labor)) OR (work N1 life N1 balanc*) OR (work* N1 load*) OR (Work* N1 Scheduling) OR (Work* N1 place*) OR (Work-related N1 Stress*) OR Absenteeism* OR Employabilit* OR Employment* OR jobsite* OR Underemploy* OR Unemploy* OR Workload OR workplace* OR worksite*)

S3 S1 AND S2

S4 TI ((job* OR work* OR labor* OR labour* OR occupation* OR vocation* OR profession*) N1 (literacy OR illitera*))

S5 S3 OR S4

S6 MH "Qualitative Studies+" OR MH "Field Studies" OR MH "Focus Groups" OR MH "Interviews+" OR MH "Observational Methods+" OR MH "Narratives" OR MH "Audiorecording" OR ZT "interview" or ZT "personal narrative" OR TI (((thematic OR content) N1 analys*) OR interview* OR (focus N1 group*) OR ethnograph* OR ethnograf* OR etnograf* OR (field N1 stud*) OR phenomenolog* OR narration* OR narrative OR (qualitative N1 (stud* OR analys* OR research* OR method*)) OR multimethodolog* OR (mixed N1 method*) OR observation* OR (grounded N1 theory) OR ((audio OR tape) N1 recording*) OR audiotape* OR ((“semi-structured” OR semistructured OR unstructured OR informal OR “in-depth” OR indepth OR face-to-face OR structured OR guide*) N3 (interview* OR discussion* OR questionnaire*))) OR AB (((thematic OR content) N1 analys*) OR (focus N1 group*) OR ethnograph* OR ethnograf* OR etnograf* OR (field N1 stud*) OR phenomenolog* OR narration* OR narrative OR (qualitative N1 (stud* OR analys* OR research* OR method*)) OR multimethodolog* OR (mixed N1 method*) OR observation* OR (grounded N1 theory) OR ((audio OR tape) N1 recording*) OR audiotape* OR ((“semi-structured” OR semistructured OR unstructured OR informal OR “in-depth” OR indepth OR face-to-face OR structured OR guide*) N3 (interview* OR discussion* OR questionnaire*))) OR SU (((thematic OR content) N1 analys*) OR (focus N1 group*) OR ethnograph* OR ethnograf* OR etnograf* OR (field N1 stud*) OR phenomenolog* OR narration* OR narrative OR (qualitative N1 (stud* OR analys* OR research* OR method*)) OR multimethodolog* OR (mixed N1 method*) OR observation* OR (grounded N1 theory) OR ((audio OR tape) N1 recording*) OR audiotape* OR ((“semi-structured” OR semistructured OR unstructured OR informal OR “in-depth” OR indepth OR face-to-face OR structured OR guide*) N3 (interview* OR discussion* OR questionnaire*)))

S7 S5 AND S6

**Embase (via Ovid)**

S1 dyslexia/ OR alexia/ OR (Dyslexi* OR alexia* OR alexic* OR (Word ADJ1 Blind*) OR (Reading ADJ1 Disorder*) OR (Reading ADJ1 Disabilit*) OR (Learning ADJ1 Disabilit*) OR (Academic ADJ1 Disabilit*) OR (Learning ADJ1 Disorder*) OR (Learning ADJ1 Disturbance*) OR (Reading ADJ1 (skill* OR ability*)) OR (Spelling ADJ1 disorder*) OR (reading ADJ1 difficult*) OR (reading ADJ1 problem*) OR (reading ADJ1 impairment*) OR (Learning ADJ1 difficult*)).ti,ab,kw.

S2 work/ or exp absenteeism/ or exp job experience/ or exp job performance/ or exp job stress/ or exp work experience/ or exp work-life balance/ or exp workload/ or exp workplace/ OR job characteristics/ OR job security/ OR work schedule/ OR job satisfaction/ OR "quality of working life"/ OR work-life balance/ OR vocational rehabilitation/ OR employment status/ or exp unemployment/ OR employability/ OR self employment/ OR supported employment/ OR occupation/ OR medical leave/ OR job finding/ OR absenteeism/ OR ((career ADJ1 choice*) OR ((Disability OR sick) ADJ1 Leave*) OR (employment ADJ1 status) OR ((sick* OR Illness) ADJ1 Day*) OR ((job* OR work* OR labor* OR labour* OR occupation* OR vocation* OR profession*) ADJ1 (site* OR status OR stress* OR applicat* OR particip* OR location OR satisf* OR performance* OR characteristic* OR accommodation* OR experience* OR skill* OR security* OR rehab*)) OR (Job-related ADJ1 Stress*) OR (Qualit* ADJ1 Work* ADJ1 Life) OR ((temporary OR “Part-time”) ADJ1 (Job* OR Employment OR work* OR labour OR labor)) OR (work ADJ1 life ADJ1 balanc*) OR (work* ADJ1 load*) OR (Work* ADJ1 Scheduling) OR (Work* ADJ1 place*) OR (Work-related ADJ1 Stress*) OR Absenteeism* OR Employabilit* OR Employment* OR jobsite* OR Underemploy* OR Unemploy* OR Workload OR workplace* OR worksite*).ti,ab,kw. OR (Job OR jobs OR labor* OR labour* OR Vocational* OR Work).ti.

S3 S1 AND S2

S4 ((job* OR work* OR labor* OR labour* OR occupation* OR vocation* OR profession*) ADJ1 (literacy OR illitera*)).ti.

S5 S3 OR S4

S6 exp qualitative research/ OR exp interview/ OR exp narrative/ OR exp storytelling/ OR exp grounded theory/ OR exp observational study/ OR exp recording/ OR exp thematic analysis/ OR exp content analysis/ OR exp ethnographic research/ OR exp field study/ OR exp participant observation/ OR exp phenomenology/ OR exp qualitative analysis/ OR exp qualitative methods/ OR exp in depth interview/ OR exp face to face interview/ OR (((thematic OR content) ADJ1 analys*) OR interview* OR (focus ADJ1 group*) OR ethnograph* OR ethnograf* OR etnograf* OR (field ADJ1 stud*) OR phenomenolog* OR narration* OR narrative OR (qualitative ADJ1 (stud* OR analys* OR research* OR method*)) OR multimethodolog* OR (mixed ADJ1 method*) OR observation* OR (grounded ADJ1 theory) OR ((audio OR tape) ADJ1 recording*) OR audiotape* OR ((“semi-structured” OR semistructured OR unstructured OR informal OR “in-depth” OR indepth OR face-to-face OR structured OR guide*) ADJ3 (interview* OR discussion* OR questionnaire*))).ti,ab,kw.

S7 S5 AND S6

S8 limit 7 to conference abstract status

S9 S7 NOT S8

**ERIC (via Ebsco)**

S1 DE "Dyslexia" OR DE "Learning Disabilities" OR DE "Reading Difficulties" OR DE "Reading Ability" OR DE "Reading Skills" OR TI (Dyslexi* OR alexia* OR alexic* OR (Word N1 Blind*) OR (Reading N1 Disorder*) OR (Reading N1 Disabilit*) OR (Learning N1 Disabilit*) OR (Academic N1 Disabilit*) OR (Learning N1 Disorder*) OR (Learning N1 Disturbance*) OR (Reading N1 (skill* OR ability*)) OR (Spelling N1 disorder*) OR (reading N1 difficult*) OR (reading N1 problem*) OR (reading N1 impairment*) OR (Learning N1 difficult*)) OR AB (Dyslexi* OR alexia* OR alexic* OR (Word N1 Blind*) OR (Reading N1 Disorder*) OR (Reading N1 Disabilit*) OR (Learning N1 Disabilit*) OR (Academic N1 Disabilit*) OR (Learning N1 Disorder*) OR (Learning N1 Disturbance*) OR (Reading N1 (skill* OR ability*)) OR (Spelling N1 disorder*) OR (reading N1 difficult*) OR (reading N1 problem*) OR (reading N1 impairment*) OR (Learning N1 difficult*)) OR SU (Dyslexi* OR alexia* OR alexic* OR (Word N1 Blind*) OR (Reading N1 Disorder*) OR (Reading N1 Disabilit*) OR (Learning N1 Disabilit*) OR (Academic N1 Disabilit*) OR (Learning N1 Disorder*) OR (Learning N1 Disturbance*) OR (Reading N1 (skill* OR ability*)) OR (Spelling N1 disorder*) OR (reading N1 difficult*) OR (reading N1 problem*) OR (reading N1 impairment*) OR (Learning N1 difficult*))

S2 DE "Job Skills" OR DE "Job Performance" OR DE "Job Satisfaction" OR DE "Employment Opportunities" OR DE "Work Environment" OR DE "Career Readiness" OR DE "Workplace Learning" OR DE "Temporary Employment" OR DE "Occupations" OR DE "Employment" OR DE "Work Experience" OR DE "Work Experience Programs" OR DE "Work Life Expectancy" OR DE "Vocational Rehabilitation" OR DE "Unemployment" OR DE "Structural Unemployment" OR DE "Employment Patterns" OR DE "Employment Problems" OR DE "Underemployment" OR DE "Job Application" OR DE "Career Choice" OR DE "Employee Absenteeism" OR TI ((career N1 choice*) OR ((Disability OR sick) N1 Leave*) OR (employment N1 status) OR ((sick* OR Illness) N1 Day*) OR ((job* OR work* OR labor* OR labour* OR occupation* OR vocation* OR profession*) N1 (site* OR status OR stress* OR applicat* OR particip* OR location OR satisf* OR performance* OR characteristic* OR accommodation* OR experience* OR skill* OR security* OR rehab*)) OR (Job-related N1 Stress*) OR (Qualit* N1 Work* N1 Life) OR ((temporary OR “Part-time”) N1 (Job* OR Employment OR work* OR labour OR labor)) OR (work N1 life N1 balanc*) OR (work* N1 load*) OR (Work* N1 Scheduling) OR (Work* N1 place*) OR (Work-related N1 Stress*) OR Absenteeism* OR Employabilit* OR Employment* OR Job OR jobs OR jobsite* OR labor* OR labour* OR Underemploy* OR Unemploy* OR Vocational* OR Work OR Workload OR workplace* OR worksite*) OR AB ((career N1 choice*) OR ((Disability OR sick) N1 Leave*) OR (employment N1 status) OR ((sick* OR Illness) N1 Day*) OR ((job* OR work* OR labor* OR labour* OR occupation* OR vocation* OR profession*) N1 (site* OR status OR stress* OR applicat* OR particip* OR location OR satisf* OR performance* OR characteristic* OR accommodation* OR experience* OR skill* OR security* OR rehab*)) OR (Job-related N1 Stress*) OR (Qualit* N1 Work* N1 Life) OR ((temporary OR “Part-time”) N1 (Job* OR Employment OR work* OR labour OR labor)) OR (work N1 life N1 balanc*) OR (work* N1 load*) OR (Work* N1 Scheduling) OR (Work* N1 place*) OR (Work-related N1 Stress*) OR Absenteeism* OR Employabilit* OR Employment* OR jobsite* OR Underemploy* OR Unemploy* OR Workload OR workplace* OR worksite*) OR SU ((career N1 choice*) OR ((Disability OR sick) N1 Leave*) OR (employment N1 status) OR ((sick* OR Illness) N1 Day*) OR ((job* OR work* OR labor* OR labour* OR occupation* OR vocation* OR profession*) N1 (site* OR status OR stress* OR applicat* OR particip* OR location OR satisf* OR performance* OR characteristic* OR accommodation* OR experience* OR skill* OR security* OR rehab*)) OR (Job-related N1 Stress*) OR (Qualit* N1 Work* N1 Life) OR ((temporary OR “Part-time”) N1 (Job* OR Employment OR work* OR labour OR labor)) OR (work N1 life N1 balanc*) OR (work* N1 load*) OR (Work* N1 Scheduling) OR (Work* N1 place*) OR (Work-related N1 Stress*) OR Absenteeism* OR Employabilit* OR Employment* OR jobsite* OR Underemploy* OR Unemploy* OR Workload OR workplace* OR worksite*)

S3 S1 AND S2

S4 DE "Workplace Literacy" OR TI ((job* OR work* OR labor* OR labour* OR occupation* OR vocation* OR profession*) N1 (literacy OR illitera*))

S5 S3 OR S4

S6 DE "Qualitative Research" OR DE "Ethnography" OR DE "Field Studies" OR DE "Focus Groups" OR DE "Grounded Theory" OR DE "Interviews" OR DE "Employment Interviews" OR DE "Semi Structured Interviews" OR DE "Structured Interviews" OR DE "Mixed Methods Research" OR DE "Naturalistic Observation" OR DE "Participant Observation" OR DE "Transcripts (Written Records)" OR DE "Personal Narratives" OR DE "Phenomenology" OR TI (((thematic OR content) N1 analys*) OR interview* OR (focus N1 group*) OR ethnograph* OR ethnograf* OR etnograf* OR (field N1 stud*) OR phenomenolog* OR narration* OR narrative OR (qualitative N1 (stud* OR analys* OR research* OR method*)) OR multimethodolog* OR (mixed N1 method*) OR observation* OR (grounded N1 theory) OR ((audio OR tape) N1 recording*) OR audiotape* OR ((“semi-structured” OR semistructured OR unstructured OR informal OR “in-depth” OR indepth OR face-to-face OR structured OR guide*) N3 (interview* OR discussion* OR questionnaire*))) OR AB (((thematic OR content) N1 analys*) OR (focus N1 group*) OR ethnograph* OR ethnograf* OR etnograf* OR (field N1 stud*) OR phenomenolog* OR narration* OR narrative OR (qualitative N1 (stud* OR analys* OR research* OR method*)) OR multimethodolog* OR (mixed N1 method*) OR observation* OR (grounded N1 theory) OR ((audio OR tape) N1 recording*) OR audiotape* OR ((“semi-structured” OR semistructured OR unstructured OR informal OR “in-depth” OR indepth OR face-to-face OR structured OR guide*) N3 (interview* OR discussion* OR questionnaire*))) OR SU (((thematic OR content) N1 analys*) OR (focus N1 group*) OR ethnograph* OR ethnograf* OR etnograf* OR (field N1 stud*) OR phenomenolog* OR narration* OR narrative OR (qualitative N1 (stud* OR analys* OR research* OR method*)) OR multimethodolog* OR (mixed N1 method*) OR observation* OR (grounded N1 theory) OR ((audio OR tape) N1 recording*) OR audiotape* OR ((“semi-structured” OR semistructured OR unstructured OR informal OR “in-depth” OR indepth OR face-to-face OR structured OR guide*) N3 (interview* OR discussion* OR questionnaire*)))

S7 S5 AND S6

**PsycInfo (via Ebsco)**

S1 DE "Alexia" OR DE "Dyslexia" OR DE "Reading Disabilities" OR TI (Dyslexi* OR alexia* OR alexic* OR (Word N1 Blind*) OR (Reading N1 Disorder*) OR (Reading N1 Disabilit*) OR (Learning N1 Disabilit*) OR (Academic N1 Disabilit*) OR (Learning N1 Disorder*) OR (Learning N1 Disturbance*) OR (Reading N1 (skill* OR ability*)) OR (Spelling N1 disorder*) OR (reading N1 difficult*) OR (reading N1 problem*) OR (reading N1 impairment*) OR (Learning N1 difficult*)) OR AB (Dyslexi* OR alexia* OR alexic* OR (Word N1 Blind*) OR (Reading N1 Disorder*) OR (Reading N1 Disabilit*) OR (Learning N1 Disabilit*) OR (Academic N1 Disabilit*) OR (Learning N1 Disorder*) OR (Learning N1 Disturbance*) OR (Reading N1 (skill* OR ability*)) OR (Spelling N1 disorder*) OR (reading N1 difficult*) OR (reading N1 problem*) OR (reading N1 impairment*) OR (Learning N1 difficult*)) OR SU (Dyslexi* OR alexia* OR alexic* OR (Word N1 Blind*) OR (Reading N1 Disorder*) OR (Reading N1 Disabilit*) OR (Learning N1 Disabilit*) OR (Academic N1 Disabilit*) OR (Learning N1 Disorder*) OR (Learning N1 Disturbance*) OR (Reading N1 (skill* OR ability*)) OR (Spelling N1 disorder*) OR (reading N1 difficult*) OR (reading N1 problem*) OR (reading N1 impairment*) OR (Learning N1 difficult*))

S2 DE "Job Characteristics" OR DE "Job Security" OR DE "Work Load" OR DE "Work Scheduling" OR DE "Job Satisfaction" OR DE "Job Performance" OR DE "Career Change" OR DE "Quality of Work Life" OR DE "Employee Efficiency" OR DE "Employee Productivity" OR DE "Work Load" OR DE "Work-Life Balance" OR DE "Occupational Status" OR DE "Vocational Rehabilitation" OR DE "Occupational Choice" OR DE "Vocational Maturity" OR DE "Employment Status" OR DE "Employability" OR DE "Employment History" OR DE "Reemployment" OR DE "Self-Employment" OR DE "Unemployment" OR DE "Supported Employment" OR DE "Occupations" OR DE "Job Characteristics" OR DE "Job Search" OR DE "Nontraditional Careers" OR DE "Occupational Choice" OR DE "Occupational Mobility" OR DE "Occupational Tenure" OR DE "Occupational Stress" OR DE "Employee Absenteeism" OR TI ((career N1 choice*) OR ((Disability OR sick) N1 Leave*) OR (employment N1 status) OR ((sick* OR Illness) N1 Day*) OR ((job* OR work* OR labor* OR labour* OR occupation* OR vocation* OR profession*) N1 (site* OR status OR stress* OR applicat* OR particip* OR location OR satisf* OR performance* OR characteristic* OR accommodation* OR experience* OR skill* OR security* OR rehab*)) OR (Job-related N1 Stress*) OR (Qualit* N1 Work* N1 Life) OR ((temporary OR “Part-time”) N1 (Job* OR Employment OR work* OR labour OR labor)) OR (work N1 life N1 balanc*) OR (work* N1 load*) OR (Work* N1 Scheduling) OR (Work* N1 place*) OR (Work-related N1 Stress*) OR Absenteeism* OR Employabilit* OR Employment* OR Job OR jobs OR jobsite* OR labor* OR labour* OR Underemploy* OR Unemploy* OR Vocational* OR Work OR Workload OR workplace* OR worksite*) OR AB ((career N1 choice*) OR ((Disability OR sick) N1 Leave*) OR (employment N1 status) OR ((sick* OR Illness) N1 Day*) OR ((job* OR work* OR labor* OR labour* OR occupation* OR vocation* OR profession*) N1 (site* OR status OR stress* OR applicat* OR particip* OR location OR satisf* OR performance* OR characteristic* OR accommodation* OR experience* OR skill* OR security* OR rehab*)) OR (Job-related N1 Stress*) OR (Qualit* N1 Work* N1 Life) OR ((temporary OR “Part-time”) N1 (Job* OR Employment OR work* OR labour OR labor)) OR (work N1 life N1 balanc*) OR (work* N1 load*) OR (Work* N1 Scheduling) OR (Work* N1 place*) OR (Work-related N1 Stress*) OR Absenteeism* OR Employabilit* OR Employment* OR jobsite* OR Underemploy* OR Unemploy* OR Workload OR workplace* OR worksite*) OR SU ((career N1 choice*) OR ((Disability OR sick) N1 Leave*) OR (employment N1 status) OR ((sick* OR Illness) N1 Day*) OR ((job* OR work* OR labor* OR labour* OR occupation* OR vocation* OR profession*) N1 (site* OR status OR stress* OR applicat* OR particip* OR location OR satisf* OR performance* OR characteristic* OR accommodation* OR experience* OR skill* OR security* OR rehab*)) OR (Job-related N1 Stress*) OR (Qualit* N1 Work* N1 Life) OR ((temporary OR “Part-time”) N1 (Job* OR Employment OR work* OR labour OR labor)) OR (work N1 life N1 balanc*) OR (work* N1 load*) OR (Work* N1 Scheduling) OR (Work* N1 place*) OR (Work-related N1 Stress*) OR Absenteeism* OR Employabilit* OR Employment* OR jobsite* OR Underemploy* OR Unemploy* OR Workload OR workplace* OR worksite*)

S3 S1 AND S2

S4 TI ((job* OR work* OR labor* OR labour* OR occupation* OR vocation* OR profession*) N1 (literacy OR illitera*))

S5 S3 OR S4

S6 DE "Qualitative Methods" OR DE "Focus Group" OR DE "Grounded Theory" OR DE "Interpretative Phenomenological Analysis" OR DE "Narrative Analysis" OR DE "Semi-Structured Interview" OR DE "Thematic Analysis" OR DE "Interviews" OR DE "Focus Group Interview" OR DE "Intake Interview" OR DE "Interview Schedules" OR DE "Job Applicant Interviews" OR DE "Psychodiagnostic Interview" OR DE "Semi-Structured Interview" OR DE "Mixed Methods Research" OR DE "Observation Methods" OR DE "Direct Observation" OR DE "Participant Observation" OR DE "Phenomenology" OR DE "Narratives" OR DE "Audiotapes" OR TI (((thematic OR content) N1 analys*) OR interview* OR (focus N1 group*) OR ethnograph* OR ethnograf* OR etnograf* OR (field N1 stud*) OR phenomenolog* OR narration* OR narrative OR (qualitative N1 (stud* OR analys* OR research* OR method*)) OR multimethodolog* OR (mixed N1 method*) OR observation* OR (grounded N1 theory) OR ((audio OR tape) N1 recording*) OR audiotape* OR ((“semi-structured” OR semistructured OR unstructured OR informal OR “in-depth” OR indepth OR face-to-face OR structured OR guide*) N3 (interview* OR discussion* OR questionnaire*))) OR AB (((thematic OR content) N1 analys*) OR (focus N1 group*) OR ethnograph* OR ethnograf* OR etnograf* OR (field N1 stud*) OR phenomenolog* OR narration* OR narrative OR (qualitative N1 (stud* OR analys* OR research* OR method*)) OR multimethodolog* OR (mixed N1 method*) OR observation* OR (grounded N1 theory) OR ((audio OR tape) N1 recording*) OR audiotape* OR ((“semi-structured” OR semistructured OR unstructured OR informal OR “in-depth” OR indepth OR face-to-face OR structured OR guide*) N3 (interview* OR discussion* OR questionnaire*))) OR SU (((thematic OR content) N1 analys*) OR (focus N1 group*) OR ethnograph* OR ethnograf* OR etnograf* OR (field N1 stud*) OR phenomenolog* OR narration* OR narrative OR (qualitative N1 (stud* OR analys* OR research* OR method*)) OR multimethodolog* OR (mixed N1 method*) OR observation* OR (grounded N1 theory) OR ((audio OR tape) N1 recording*) OR audiotape* OR ((“semi-structured” OR semistructured OR unstructured OR informal OR “in-depth” OR indepth OR face-to-face OR structured OR guide*) N3 (interview* OR discussion* OR questionnaire*)))

S7 S5 AND S6

**PubMed**

S1 dyslexia[MeSH] OR "Learning Disabilities"[Mesh] OR Dyslexi*[tiab] OR alexia*[tiab] OR alexic*[tiab] OR Word Blind*[tiab] OR Reading Disorder*[tiab] OR Reading Disabilit*[tiab] OR Learning Disabilit*[tiab] OR Academic Disabilit*[tiab] OR Learning Disorder*[tiab] OR Learning Disturbance*[tiab] OR Reading skill*[tiab] OR Spelling disorder*[tiab] OR reading difficult*[tiab] OR reading problem*[tiab] OR reading impairment*[tiab] OR Learning difficult*[tiab]

S2 "Employment"[Mesh] OR "Work"[Mesh] OR "Job Application"[Mesh] OR "Job Satisfaction"[Mesh] OR "Occupations"[Mesh] OR "Occupational Stress"[Mesh] OR "Career Choice"[Mesh] OR absenteeism[MeSH] OR "Sick Leave"[Mesh] OR "Work-Life Balance"[Mesh] OR "Workload"[Mesh] OR Disability Leave*[tiab] OR Employabilit*[tiab] OR employment status[tiab] OR Employment*[tiab] OR Illness Day*[tiab] OR Job[ti] OR Jobs[ti] OR Job Performance*[tiab] OR Job Characteristic*[tiab] OR Job Accommodation*[tiab] OR Job Experience*[tiab] OR Job Skill*[tiab] OR Job Securit*[tiab] OR Job applicat*[tiab] OR job site*[tiab] OR job status[tiab] OR job stress*[tiab] OR Job-related Stress*[tiab] OR jobsite*[tiab] OR labor*[ti] OR labour*[ti] OR part time job*[tiab] OR parttime job*[tiab] OR part time work*[tiab] OR parttime work*[tiab] OR Occupational Status[tiab] OR occupational status[tiab] OR Occupational Stress*[tiab] OR Professional Stress*[tiab] OR Quality of Working Life*[tiab] OR Sick Day*[tiab] OR sick leave*[tiab] OR temporary job*[tiab] OR temporary work*[tiab] OR Underemploy*[tiab] OR Unemploy*[tiab] OR Vocation*[tiab] OR Work life balance*[tiab] OR Workload*[tiab] OR Work load*[tiab] OR Work Location*[tiab] OR work particip*[tiab] OR work place*[tiab] OR work scheduling[tiab] OR work site*[tiab] OR Work status[tiab] OR work[ti] OR working status[tiab] OR workplace*[tiab] OR Work-related Stress*[tiab] OR worksite*[tiab]

S3 "Qualitative Research"[Mesh] OR "Focus Groups"[Mesh] OR "Interview" [Publication Type] OR "Interviews as Topic"[Mesh] OR "Narration"[Mesh] OR "Personal Narratives as Topic"[Mesh] OR "Grounded Theory"[Mesh] OR "Observational Studies as Topic"[Mesh] OR "Observational Study" [Publication Type] OR "Tape Recording"[Mesh] OR thematic analys*[tiab] OR content analys*[tiab] OR focus group*[tiab] OR ethnograph*[tiab] OR ethnograf*[tiab] OR etnograf*[tiab] OR field stud*[tiab] OR phenomenolog*[tiab] OR narration*[tiab] OR narrative[tiab] OR qualitative stud*[tiab] OR qualitative analys*[tiab] OR qualitative research*[tiab] OR qualitative method*[tiab] OR multimethodolog*[tiab] OR mixed method*[tiab] OR observation*[tiab] OR grounded theory[tiab] OR audio recording*[tiab] OR tape recording*[tiab] OR audiotape*[tiab] OR ((semi-structured[tiab] OR semistructured[tiab] OR unstructured[tiab] OR informal[tiab] OR in-depth[tiab] OR indepth[tiab] OR face-to-face[tiab] OR structured[tiab] OR guide*[tiab]) AND (interview*[tiab] OR discussion*[tiab] OR questionnaire*[tiab]))

S4 S1 AND S2 AND S3

**Web of Science**

S1 TS=(Dyslexi* OR alexia* OR alexic* OR (Word NEAR/1 Blind*) OR (Reading NEAR/1 Disorder*) OR (Reading NEAR/1 Disabilit*) OR (Learning NEAR/1 Disabilit*) OR (Academic NEAR/1 Disabilit*) OR (Learning NEAR/1 Disorder*) OR (Learning NEAR/1 Disturbance*) OR (Reading NEAR/1 (skill* OR ability*)) OR (Spelling NEAR/1 disorder*) OR (reading NEAR/1 difficult*) OR (reading NEAR/1 problem*) OR (reading NEAR/1 impairment*) OR (Learning NEAR/1 difficult*))

S2 TS=((career NEAR/1 choice*) OR ((Disability OR sick) NEAR/1 Leave*) OR (employment NEAR/1 status) OR ((sick* OR Illness) NEAR/1 Day*) OR ((job* OR work* OR labor* OR labour* OR occupation* OR vocation* OR profession*) NEAR/1 (site* OR status OR stress* OR applicat* OR particip* OR location OR satisf* OR performance* OR characteristic* OR accommodation* OR experience* OR skill* OR security* OR rehab*)) OR (Job-related NEAR/1 Stress*) OR (Qualit* NEAR/1 Work* NEAR/1 Life) OR ((temporary OR “Part-time”) NEAR/1 (Job* OR Employment OR work* OR labour OR labor)) OR (work NEAR/1 life NEAR/1 balanc*) OR (work* NEAR/1 load*) OR (Work* NEAR/1 Scheduling) OR (Work* NEAR/1 place*) OR (Work-related NEAR/1 Stress*) OR Absenteeism* OR Employabilit* OR Employment* OR jobsite* OR Underemploy* OR Unemploy* OR Workload OR workplace* OR worksite*)

S3 TI=(Job OR jobs OR labor* OR labour* OR Vocational* OR “Work”)

S4 S2 OR S3

S5 S1 AND S4

S6 TI=((job* OR work* OR labor* OR labour* OR occupation* OR vocation* OR profession*) NEAR/1 (literacy OR illitera*))

S7 S5 OR S6

S8 TS=(((thematic OR content) NEAR/1 analys*) OR interview* OR (focus NEAR/1 group*) OR ethnograph* OR ethnograf* OR etnograf* OR (field NEAR/1 stud*) OR phenomenolog* OR narration* OR narrative OR (qualitative NEAR/1 (stud* OR analys* OR research* OR method*)) OR multimethodolog* OR (mixed NEAR/1 method*) OR observation* OR (grounded NEAR/1 theory) OR ((audio OR tape) NEAR/1 recording*) OR audiotape* OR ((“semi-structured” OR semistructured OR unstructured OR informal OR “in-depth” OR indepth OR face-to-face OR structured OR guide*) NEAR/3 (interview* OR discussion* OR questionnaire*)))

S9 S7 AND S8
